# Diffuse Alveolar Hemorrhage Induced by Vaping

**DOI:** 10.1155/2018/9724530

**Published:** 2018-06-07

**Authors:** Michael Agustin, Michele Yamamoto, Felix Cabrera, Ricardo Eusebio

**Affiliations:** Guam Regional Medical City, 133 Route 3, Dededo, Guam 96929, USA

## Abstract

There has been a significant increase in electronic cigarette (e-cigarette) use since its introduction in 2007. Ironically, there remains very few published literature on the respiratory complications of e-cigarettes. The use of personalized vaporizers or commonly known as “vaping” has started to overtake standard e-cigarette. Its dynamic vaporizer customization makes it challenging to assess long-term health effects. Case reports on the pulmonary complications of e-cigarettes are limited to bronchiectasis, eosinophilic pneumonia, pleural effusion, and suspected hypersensitivity pneumonitis. Diffuse alveolar hemorrhage (DAH) is bleeding into the alveolar spaces of the lung secondary to disruption of the alveolar-capillary basement membrane. We report a case of young male presenting with subacute respiratory failure. He was later found to have diffuse alveolar hemorrhage syndrome that is likely induced by aggressive vaping. This adds up to the rising concern on the possible serious complications of this innovative technology designed as a safer alternative to traditional cigarettes.

## 1. Introduction 

Electronic cigarettes (e-cigarettes) were initially marketed in 2007 and since then there had been a steady growth of its use amongst naive and chronic smokers [1]. “Vapor” is the aerosol inhaled through heating a liquid in the device [2]. Nowadays, personal vaporizers or “vape” are considered a step-up from the standard e-cigarette. Its popularity is mainly driven by the ability for device customization. Users can mix and match liquids to achieve preferred flavor and cloud production.

To date, there still varied results on how e-cigarette affects smoking cessation [3–5]. Whether or not the efficacy of e-cigarettes for assisting smoking cessation becomes established, its safety profile and health related risks warrant extensive evaluation. The dynamic device design makes it difficult to perform calculations of the harm compared with conventional cigarette smoking [6].

There exists very few reported cases of pulmonary complications of e-cigarettes and these are limited to hypersensitivity inhalational injury, pneumonitis, and acute eosinophilic pneumonia [7]. We report a case of diffuse alveolar hemorrhage that we believe was caused by aggressive use of personalized vaporizer. This aims to site the serious complication of vaping despite being marketed as a safe substitute to conventional smoking.

## 2. Case Presentation

**Figure 1:**
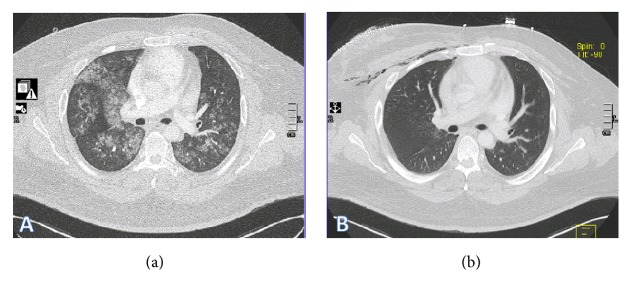
(a) Chest CT scan on admission with diffuse ground glass opacity and bilateral patchy consolidation. (b) Chest CT scan two weeks after admission showing improvement of parenchymal lesions. Patient developed mild subcutaneous emphysema following right lung wedge resection biopsy.

**Figure 2:**
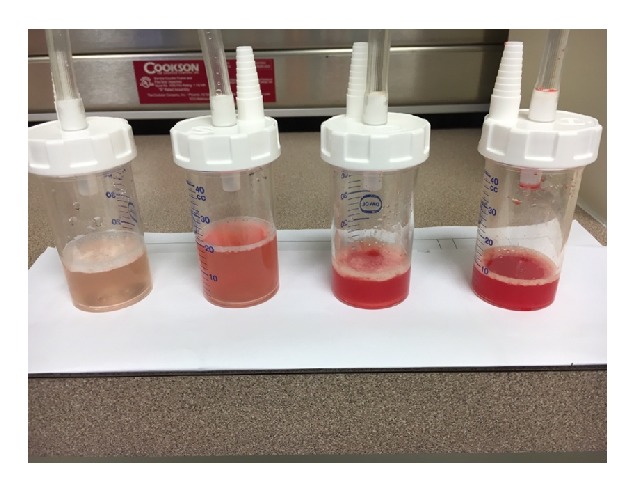
Bronchoalveolar lavage (BAL) revealed increasing blood in four sequential aliquots confirming diagnosis of diffuse alveolar hemorrhage (DAH).

**Figure 3:**
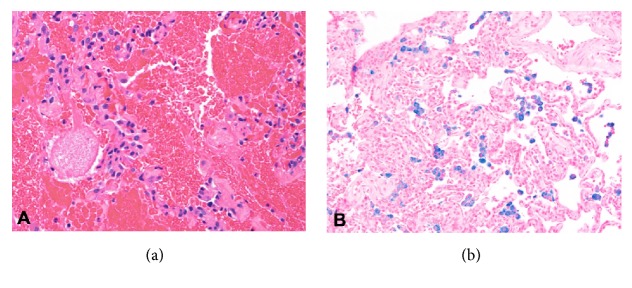
(a) Lung biopsy showing alveolar hemorrhage. (b) Prussian blue iron staining reflecting old hemorrhage.

Thirty-three-year-old male with diabetes and seizure disorder presented to the emergency department (ED) with worsening dyspnea and hemoptysis. Two weeks prior to his ED presentation, he was treated with antibiotics for community acquired pneumonia with minimal improvement. Upon further inquiry, patient admitted to vaping for the past 2 months with overtly increased exposure time and has experimented on new flavors. He denied previous or current recreational drug use. CT scan of the chest showed diffuse ground glass opacities and bilateral patchy consolidation (Figure 1(a)). He had worsening hypoxia that required noninvasive ventilation. His echocardiogram was otherwise normal. Bronchoscopic examination failed to demonstrate airway lesions. Bronchoalveolar lavage (BAL) revealed increasing blood in four sequential aliquots confirming diagnosis of DAH (Figure 2). BAL cell count showed greater than 30,000 RBCs and 800 WBCs, 42% neutrophils, 36% lymphocytes, 1% eosinophils, and 21% macrophages. All inflammatory serologies were unremarkable: erythrocyte sedimentation rate (ESR), C-reactive protein (CRP), rheumatoid factor (RF), antinuclear antibody (ANA), and anti-antineutrophil cytoplasmic antibodies (ANCA). In addition, serum eosinophil count, anti-glomerular basement membrane (GBM) antibodies, and anti-phospholipid antibodies were all normal. Urine toxicology screen which includes amphetamines, cannabinoids, and cocaine was negative. There was no microbiologic growth on all BAL specimens. Patient was treated with pulse dose steroids after DAH was confirmed with BAL aliquots (Figure 2). He underwent right wedge resection lung biopsy which revealed evidence of bland pulmonary hemorrhage (Figure 3(a)) with no evidence of capillaritis or diffuse alveolar damage (DAD). Prussian blue iron staining was also noted which reflects old hemorrhage (Figure 3(b)). His symptoms improved with complete resolution of alveolar hemorrhage on chest CT scan after 2 weeks (Figure 1(b)). His steroids were tapered quickly and he has not used a personal vaporizer since then.

## 3. Discussion

The use of personalized vaporizers or vaping has exponentially increased replacing standard e-cigarettes. It has attracted young adults as they can customize and reuse their vaporizers making it a cheaper alternative. While vaping is likely less toxic than cigarette smoking given the lack of most combustible tobacco constituents, we are still uncertain on the health hazards of the reduced toxins. The rapidly changing designs of the product and the lack of long-term follow-up make assessment on the risk of health challenging [6].

There are a few published reports on the respiratory complications of e-cigarettes. These are limited to bronchiectasis, eosinophilic pneumonia, pleural effusion, and suspected hypersensitivity pneumonitis [7, 8]. Biopsy-proven Respiratory Bronchiolitis Interstitial Lung Disease (RBILD) has been demonstrated with e-cigarette use in one case report [9]. Atypical pneumonitis evidenced by bronchoalveolar lavage analysis and lipid staining has also been reported. The syndrome of diffuse alveolar hemorrhage is caused by injury or inflammation of the arterioles, venules, or alveolar capillaries. DAH is often a catastrophic clinical syndrome causing respiratory failure. The etiology of DAH is multifactorial and can be divided into three categories, namely, pulmonary capillaritis, bland alveolar hemorrhage, and diffuse alveolar damage (DAD) [10]. Recognition of DAH often requires BAL analysis as symptoms are nonspecific. Hemoptysis is absent in up to one-third of patients, and radiographic imaging is also nonspecific and similar to other acute alveolar filling processes [10, 11]. Lung biopsy is often required to establish definite pathologic pattern.

Cases relating to smoking-induced DAH are limited to few reports on cocaine and cannabis use [12, 13]. We postulate that direct exposure to inhalation products of heated liquid resulted to alveolar injury. The exposure may or may not elicit alveolar inflammation. Although cell culture and experimental animal data indicate that e-cigarette have the potential for inducing inflammation, this still remains unclear on human studies [14]. The e-juice used in this case had a predominant base of propylene glycol (PG) in addition to nicotine, water, and 2 artificial flavorings. The temporal relations of patient's aggressive vaping and the development of alveolar hemorrhage remain circumstantial. However, we are unaware of any other explanation for the patient's bland pulmonary hemorrhage. Cessation of implicated exposure and supportive care are core treatments of DAH. Immunosuppressive medication is added if there is histologic evidence of capillaritis. In our case, it is challenging to determine if the improvement of patient's symptoms and radiographic findings were related to discontinuation of exposure to vapor or the effect of steroids. Pulse steroids were initiated early in our patient as his hypoxia continued to worsen. This was tapered quickly as soon as inflammatory makers showed negative results. Our initial aggressive treatment was geared to decrease the high morbidity and mortality associated with DAH. In conclusion, severe complication may arise from vaping despite being marketed as a safe alternative to conventional smoking. This would encourage clinicians to be vigilant on the respiratory risks of vaping. Likewise, this should also encourage more research on the pulmonary toxicity of vaping as the surge of its use continues in the coming years.

## Data Availability

The data that support the findings of this study are available from the corresponding author upon reasonable request.
